# Peripheral insulin resistance rather than beta cell dysfunction accounts for geographical differences in impaired fasting blood glucose among sub-Saharan African individuals: findings from the RODAM study

**DOI:** 10.1007/s00125-017-4216-4

**Published:** 2017-01-31

**Authors:** Karlijn A. C. Meeks, Karien Stronks, Adebowale Adeyemo, Juliet Addo, Silver Bahendeka, Erik Beune, Ellis Owusu-Dabo, Ina Danquah, Cecilia Galbete, Peter Henneman, Kerstin Klipstein-Grobusch, Frank P. Mockenhaupt, Kwame Osei, Matthias B. Schulze, Joachim Spranger, Liam Smeeth, Charles Agyemang

**Affiliations:** 1 Department of Public Health, Academic Medical Center, University of Amsterdam, Meibergdreef 9, 1105AZ Amsterdam, the Netherlands; 2 Center for Research on Genomics and Global Health, National Human Genome Research Institute, National Institutes of Health, Bethesda, MD USA; 3 Department of Non-Communicable Disease Epidemiology, London School of Hygiene and Tropical Medicine, London, UK; 4 Mother Kevin Postgraduate Medical School (MKPGMS), Uganda Martyrs University, Kampala, Uganda; 5 Kumasi Centre for Collaborative Research, Kwame Nkrumah University of Science and Technology (KNUST), Kumasi, Ghana; 6 Department of Molecular Epidemiology, German Institute of Human Nutrition Potsdam-Rehbruecke, Nuthetal, Germany; 7 Department of Clinical Genetics, Academic Medical Center, University of Amsterdam, Amsterdam, the Netherlands; 8 Julius Global Health, Julius Center for Health Sciences and Primary Care, University Medical Center Utrecht, Utrecht, the Netherlands; 9 Division of Epidemiology and Biostatistics, School of Public Health, Faculty of Health Sciences, University of the Witwatersrand, Johannesburg, South Africa; 10 Institute of Tropical Medicine and International Health, Charité–University Medicine Berlin, Berlin, Germany; 11 Division of Endocrinology, Diabetes and Metabolism, The Ohio State University Wexner Medical Center, Columbus, OH USA; 12 Department of Endocrinology and Metabolism, Charité–University Medicine Berlin, Berlin, Germany; 13 German Centre for Cardiovascular Research (DZHK), Berlin, Germany; 14 Center for Cardiovascular Research (CCR), Charité–University Medicine Berlin, Berlin, Germany

**Keywords:** Beta cell function, Impaired fasting glycaemia, Insulin resistance, RODAM study, Sub-Saharan Africans, Type 2 diabetes

## Abstract

**Aims/hypothesis:**

The aim of this study was to assess the extent to which insulin resistance and beta cell dysfunction account for differences in impaired fasting blood glucose (IFBG) levels in sub-Saharan African individuals living in different locations in Europe and Africa. We also aimed to identify determinants associated with insulin resistance and beta cell dysfunction among this population.

**Methods:**

Data from the cross-sectional multicentre Research on Obesity and Diabetes among African Migrants (RODAM) study were analysed. Participants included Ghanaian individuals without diabetes, aged 18–96 years old, who were residing in Amsterdam (*n* = 1337), Berlin (*n* = 502), London (*n* = 961), urban Ghana (*n* = 1309) and rural Ghana (*n* = 970). Glucose and insulin were measured in fasting venous blood samples. Anthropometrics were assessed during a physical examination. Questionnaires were used to assess demographics, physical activity, smoking status, alcohol consumption and energy intake. Insulin resistance and beta cell function were determined using homeostatic modelling (HOMA-IR and HOMA-B, respectively). Logistic regression analysis was used to study the contribution of HOMA-IR and inverse HOMA-B (beta cell dysfunction) to geographical differences in IFBG (fasting glucose 5.6–6.9 mmol/l). Multivariate linear regression analysis was used to identify determinants associated with HOMA-IR and inverse HOMA-B.

**Results:**

IFBG was more common in individuals residing in urban Ghana (OR 1.41 [95% CI 1.08, 1.84]), Amsterdam (OR 3.44 [95% CI 2.69, 4.39]) and London (OR 1.58 [95% CI 1.20 2.08), but similar in individuals living in Berlin (OR 1.00 [95% CI 0.70, 1.45]), compared with those in rural Ghana (reference population). The attributable risk of IFBG per 1 SD increase in HOMA-IR was 69.3% and in inverse HOMA-B was 11.1%. After adjustment for HOMA-IR, the odds for IFBG reduced to 0.96 (95% CI 0.72, 1.27), 2.52 (95%CI 1.94, 3.26) and 1.02 (95% CI 0.78, 1.38) for individuals in Urban Ghana, Amsterdam and London compared with rural Ghana, respectively. In contrast, adjustment for inverse HOMA-B had very minor impact on the ORs of IFBG. In multivariate analyses, BMI (β = 0.17 [95% CI 0.11, 0.24]) and waist circumference (β = 0.29 [95%CI 0.22, 0.36]) were most strongly associated with higher HOMA-IR, whereas inverse HOMA-B was most strongly associated with age (β = 0.20 [95% CI 0.16, 0.23]) and excess alcohol consumption (β = 0.25 [95% CI 0.07, 0.43]).

**Conclusions/interpretation:**

Our findings suggest that insulin resistance, rather than beta cell dysfunction, is more important in accounting for the geographical differences in IFBG among sub-Saharan African individuals. We also show that BMI and waist circumference are important factors in insulin resistance in this population.

## Introduction

The burden of type 2 diabetes is globally high, with sub-Saharan African migrants in Europe being almost three times more likely to have type 2 diabetes than the European host population [1]. In the sub-Saharan African region, type 2 diabetes prevalence has been increasing rapidly over recent decades and is projected to increase most in this region over all world regions [2].

A combination of genetics and health-related behaviours are thought to cause type 2 diabetes by contributing to increased insulin resistance and reduced beta cell function. These result in impaired fasting blood glucose (IFBG) and, ultimately, type 2 diabetes [3, 4]. Interestingly, increased insulin resistance is initially compensated by elevated beta cell function [5, 6]. However, once insulin resistance increases to a level where beta cell function can no longer compensate, IFBG develops [6].

The pathogenesis of type 2 diabetes in sub-Saharan Africans is still poorly understood [3]. The importance of insulin resistance and beta cell failure to produce insulin in the development of IFBG and type 2 diabetes seems to differ among sub-Saharan African populations compared with European populations [7–12]. Studies have shown that insulin resistance is more severe and beta cell insulin production is elevated in African populations with normal glucose tolerance compared with European populations [8, 9, 11]. Furthermore, during the progression of normal glucose tolerance to IFBG and type 2 diabetes, insulin production has been found to decrease more rapidly in African populations than in European populations [10, 11]. However, most of these studies have been performed in African-Americans [8, 9, 11, 12] (who are more genetically admixed compared with sub-Saharan African populations) or on a small sample size [7, 10].

The high burden of type 2 diabetes among African migrants in Europe, and the steep increase in type 2 diabetes in urbanising Africa, indicate that environmental exposures, such as changes in health-related behaviours, and resulting changes in anthropometrics might play a role in a disruption of balance between beta cell function and insulin sensitivity in these individuals [13]. However, data on the role of the environment in the development of insulin resistance and beta cell dysfunction among Africans are limited. Furthermore, there are indications that the determinants of insulin resistance and beta cell dysfunction at play among Africans may differ from European populations [14]. However, it is unclear which determinants (e.g. sociodemographic factors, anthropometrics or health-related behaviours) are driving insulin resistance and beta cell dysfunction in African populations [15].

The Research on Obesity and Diabetes among African Migrants (RODAM) study found differences in IFBG and type 2 diabetes prevalence among Ghanaians that were resident in multiple geographical locations. While type 2 diabetes prevalence was highest among Ghanaians in Berlin (12.8%), followed by Amsterdam (11.2%), London (9.4%), urban Ghana (9.3%) and rural Ghana (4.9%), IFBG prevalence was highest among Ghanaians in Amsterdam (27.5%), followed by London (14.7%), urban Ghana (11.9%), Berlin (11.2%) and rural Ghana (11.2%) [16]. The relatively homogenous but geographically distinct population of the RODAM study offers an important opportunity to investigate the role of environment and health-related behaviours in insulin resistance and beta cell dysfunction among this sub-Saharan African population.

We therefore used data from the RODAM study as a means to: (1) assess the contribution of insulin resistance and beta cell dysfunction to differences in IFBG between sub-Saharan Africans in different geographical locations; and (2) investigate which sociodemographic, anthropometric and health-related behaviour determinants contribute to insulin resistance and beta cell dysfunction among this population.

## Methods

### Study design and participants

**Fig. 1:**
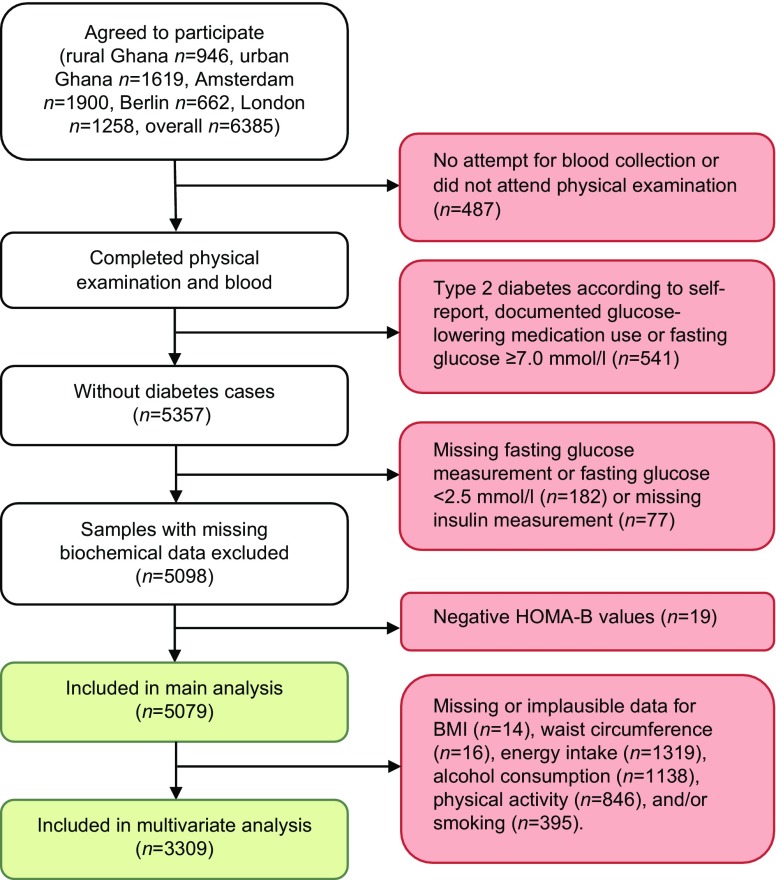
Flow chart of study design and inclusion in analysis

The design of the RODAM study and the sampling strategy have been described in detail elsewhere [17]. In brief, between 2012 and 2015 Ghanaian adult residents (aged 18–96 years old) in rural Ghana, urban Ghana, Amsterdam, London and Berlin were recruited (Fig. 1). Ghanaian origin was defined as being born in Ghana and having at least one parent born in Ghana (first generation) or not born in Ghana but having both parents born in Ghana (second generation). In Ghana, participants were randomly selected from a list of 30 enumeration areas in the Ashanti Region, based on the 2010 census. In Amsterdam, Ghanaian participants were randomly drawn from the Amsterdam Municipal Health Register, which holds data on country of birth of citizens and their parents. In London, Ghanaian organizations served as the sampling frame since there was no population register for migrant groups. In Berlin, a similar approach to that in London was used, including member lists of Ghanaian churches and organizations as the sampling frame. In the European sites, selected participants were sent study information and a response card that they could use to opt in via mail.

Overall 6385 participants were recruited. Of those invited to participate, the participation rate was 76% in rural Ghana, 74% in urban Ghana, 75% in London and 68% in Berlin. In Amsterdam, a response to the invitation to participate was received from 67% and, of these, 53% agreed to participate.

Data from participants who had attended their physical examination at a research location and had at least one attempt for blood collection were included in the analysis (Fig. 1). For the current analysis participants with diabetes according to self-report, documented as using glucose-lowering medication or with a fasting glucose ≥7.0 mmol/l were excluded from the analyses. This was carried out mainly to eliminate treatment effect. In addition, samples with plasma glucose of <2.5 mmol/l were assumed to belong to individuals that suffer from hypoglycaemia, which is a non-steady-state situation, or reflect an assay problem [18] and were, therefore, excluded from analyses. Furthermore, those with missing fasting glucose or insulin measurements and with negative HOMA-B values were also excluded. In total 5079 participants were included in the overall analysis. After exclusion of missing and implausible data, such as energy intake >95^th^ percentile (20,658 kJ/day), 3309 participants remained for the multivariate analyses. The main analysis, which assessed the contribution of insulin resistance and beta cell dysfunction to geographical differences in IFBG, had an achieved power of 0.99, detecting the reported differences in odds for IFBG between locations at an α of 0.05.

Ethics committees of the institutions involved in Ghana, the Netherlands, the UK and Germany approved the study protocols before data collection commenced in each country. Written informed consent was obtained from all participants enrolled in the study.

### Measurements

Data collection was standardised for all locations including equipment, transport procedures, operating procedures and questionnaires.

#### Demographics, education and health-related behaviours

The questionnaire for participants’ demographics, educational level and health-related behaviours [17] was either administered by interviewer or self-administered. From this questionnaire, a positive reply to the question ‘Has someone in your immediate family (your parents, brothers, sisters, or children) been diagnosed with diabetes?’ was used to determine family history of diabetes. Smoking was assessed as a positive reply to the question ‘Do you smoke at all?’. The WHO STEPS questionnaire [19] was used to derive physical activity in metabolic equivalent (MET) h/week, which included physical activity at work, while commuting and in leisure time [20]. Energy intake was assessed by means of a standardised Food Propensity Questionnaire developed specifically for Ghanaian populations, based on the European Food Propensity Questionnaire [21]. It queried the usual frequency of food intake during the past 12 months. Alcohol consumption was calculated in units/week, with 500 ml of beer, 250 ml of wine and 80 ml of spirits being counted a 1 unit of alcohol. Alcohol consumption was subsequently classified based on the European Society of Hypertension/European Society of Cardiology (ESH/ESC) guidelines for the management of arterial hypertension [22] into ‘no alcohol consumption’, ‘within the guideline’ and ‘exceeding the guideline’.

#### Anthropometric measurements

Participants were asked to visit a research facility to take part in a physical examination, which was conducted by a trained research assistant according to standard operational procedures. During the physical examination, height, weight and waist circumference were assessed in light clothing, in duplicate and truncated to one decimal place. For height and weight measurements, a SECA 217 (SECA, Hamburg, Germany) portable stadiometer and SECA 877 scale were used, respectively. BMI was calculated by dividing weight (kg) by squared height (m^2^). Blood pressure was measured three times after 5 min of rest, in a seated position, using the Microlife WatchBP home monitor (Microlife WatchBP AG, Widnau, Switzerland); the mean of the last two measurements was used to determine systolic and diastolic blood pressure. Hypertension was defined according to the WHO definition i.e. systolic blood pressure of over 140 mmHg and/or diastolic blood pressure of over 90 mmHg, or participants receiving anti-hypertensive medication for treatment of hypertension [23].

#### Biochemical analyses

Fasting blood samples were drawn at a research location by trained research assistants according to standard operating procedures. All blood samples were processed and divided into aliquots immediately after collection, and then temporarily stored at −20°C. Subsequently, samples were transported to the local research centres and were checked, registered and stored at −80°C before being shipped to the laboratory at Charité–University Medicine Berlin (Berlin, Germany) for determination of biochemical variables.

Insulin concentrations were assessed using the Mercodia ELISA kit (Mercodia, Uppsala, Sweden). The intra-assay CV was 3.4% and the inter-assay CV 3.0%. The lower detection limit of the Mercodia ELISA was 76 pmol/l. Fasting glucose, total cholesterol, HDL-cholesterol, LDL-cholesterol and triacylglycerols were determined using the ABX Pentra 400 chemistry analyser (HORIBA ABX, Montpellier, France). Extensive quality checks were conducted during the biochemical analysis, including blinded serial measurements.

Insulin resistance and beta cell function were assessed using HOMA [24]: HOMA-derived insulin resistance index (HOMA-IR) and HOMA-derived beta cell function (HOMA-B). Both of these have been used extensively in epidemiological studies [18]. The original HOMA modelling equations were used to calculate HOMA-IR and HOMA-B [18]. IFBG was defined as a fasting glucose between 5.6 and 6.9 mmol/l, according to the American Diabetes Association (ADA) criteria [25], since previous studies have shown that a substantial proportion of the population is misclassified when using the 6.1 mmol/l blood glucose threshold [26, 27].

### Data analysis

The interaction between location and sex in relation to IFBG tested not significant, therefore analyses were combined for men and women. The characteristics of participants included in the analysis were expressed as medians with interquartile ranges, means with corresponding 95% CI or percentages with corresponding 95% CI. Mann–Whitney *U* tests were used to compare HOMA-IR and HOMA-B between locations. The age- and sex-adjusted association of HOMA-IR and HOMA-B with IFBG was studied using logistic regression analysis combining all geographical locations. In these logistic regression analyses, the inverse of HOMA-B was calculated (1/HOMA-B) and both HOMA-IR and inverse HOMA-B were converted into standardised *z* scores in order to make them comparable. Attributable risk was calculated to gain more insight into the relative contribution of HOMA-IR vs HOMA-B to IFBG; attributable risk with corresponding 95% CI was calculated for IFBG per SD increase in HOMA-IR and inverse HOMA-B, both adjusted for age and sex. In assessing the contribution of HOMA-IR and inverse HOMA-B to the geographical differences in IFBG, logistic regression analysis was carried out, using rural Ghana as reference category. In bivariate and multivariate linear regression analyses, the association between age, sex, family history of diabetes, anthropometrics, health-related behaviours and geographical location with HOMA-IR and inverse HOMA-B was studied. In these linear regression analyses, HOMA-IR and inverse HOMA-B were first multiplied by 10 and log_10_ transformed to account for non-normal distribution before they were subsequently converted to standardised *z* scores to make them comparable. All continuous determinants were also converted to *z* scores. All analyses were performed according to a complete case analysis approach using SPSS version 23 [28] and R version 3.2.3 [29].

## Results

### Characteristics of the study population

Participants in rural Ghana and London were slightly older than those in urban Ghana, Amsterdam and Berlin (Table 1). In urban Ghana, relatively more women participated than in the other locations, while in Berlin more men participated than in the other locations. Education level differed greatly between locations. London Ghanaians reported highest educational attainment, while rural Ghanaians reported the lowest. Within the European locations, Amsterdam Ghanaians reported the lowest levels of education. Family history of diabetes, BMI and waist circumference were all lowest in rural Ghana and comparable between the other locations. Compared with rural Ghana, systolic blood pressure, diastolic blood pressure, and the prevalence of hypertension were slightly higher in urban Ghana and considerably higher in the European locations. Total cholesterol and LDL-cholesterol in urban Ghana were comparable to those in European locations but higher than in rural Ghana. Triacylglycerols were higher in rural and urban Ghana than in the European locations, while HDL-cholesterol was higher in the European locations compared with Ghana. The highest physical activity levels, according to MET scores in h/week, were observed in rural Ghana, followed by Amsterdam, Berlin, urban Ghana and then London. Energy intake in kJ/day was highest in Berlin and London, followed by rural Ghana. Energy intake was lowest urban Ghana, followed by Amsterdam. The highest smoking prevalence and alcohol consumption were observed in Berlin.

**Table 1:** Characteristics of the participants included in the analysis (*n* = 5079)

Characteristics	Rural Ghana *n* = 970	Urban Ghana *n* = 1309	Amsterdam *n* = 1337	Berlin *n* = 502	London *n* = 961
Demographics					
Age (years)	47.9 (47.0, 48.8)	44.6 (43.9, 45.2)	44.6 (44.0, 45.1)	43.4 (42.4, 44.3)	46.2 (45.5, 46.9)
Male (%)	39.0 (35.9, 42.1)	28.1 (25.7, 30.6)	38.0 (35.4, 40.6)	52.2 (47.8, 56.5)	38.5 (35.5, 41.6)
Length of stay in Europe (years)	–	–	17.2 (16.7, 17.7)	15.9 (14.9, 16.9)	16.0 (15.3, 16.7)
First generation migrants (%)	–	–	97.2 (96.1, 98.0)	97.2 (95.3, 98.3)	96.6 (95.2, 97.7)
Education level (%)					
None or primary (elementary)	58.8 (55.5, 62.0)	44.1 (41.4, 46.9)	33.1 (30.5, 35.8)	7.8 (5.7, 10.6)	8.9 (7.1, 11.0)
Lower secondary	30.5 (27.6, 33.6)	37.6 (36.3, 41.7)	36.6 (34.0, 39.3)	50.9 (46.5, 55.3)	32.2 (29.1, 35.5)
Higher secondary	6.9 (5.4, 8.7)	12.3 (10.6, 14.2)	24.0 (21.7, 26.5)	28.2 (24.4, 32.2)	25.8 (22.9, 28.9)
Tertiary education	3.8 (2.7, 5.2)	4.6 (3.6, 5.9)	6.2 (5.0, 7.7)	13.1 (10.4, 16.3)	33.2 (30.0, 36.5)
Cardio-metabolic traits					
Fasting glucose (mmol/l)	5.0 (4.9, 5.0)	5.1 (5.0, 5.1)	5.2 (5.2, 5.3)	4.8 (4.7, 4.9)	5.0 (4.9, 5.0)
Insulin (pmol/l)	27.8 (25.7)	43.8 (43.8)	45.1 (36.1)	36.1 (26.4)	41.7 (35.4)
Family history of diabetes (%)	10.0 (8.2, 12.1)	18.3 (16.2, 20.5)	18.1 (15.8, 20.5)	23.4 (20.0, 27.4)	23.4 (20.6, 26.5)
BMI (kg/m^2^)	22.5 (22.2, 22.7)	26.9 (26.6, 27.2)	28.6 (28.3, 28.9)	27.3 (26.8, 27.7)	29.3 (29.0, 29.7)
Waist circumference (cm)	80.7 (80.0, 81.4)	89.0 (88.4, 89.7)	93.1 (92.4, 93.7)	90.7 (89.8, 91.7)	94.3 (93.6, 95.1)
Systolic BP (mmHg)	124.1 (122.8, 125.4)	125.8 (124.7, 126.9)	132.6 (131.6, 133.5)	134.3 (132.7, 136.0)	134.4 (133.3, 135.5)
Diastolic BP (mmHg)	76.8 (86.0, 77.5)	79.1 (78.4, 79.7)	83.5 (82.9, 84.1)	85.4 (84.4, 86.4)	83.0 (82.4, 79.7)
Hypertension (%)	26.4 (23.7, 29.3)	28.4 (26.1, 30.9)	45.9 (43.3, 48.6)	49.0 (44.6, 53.4)	48.5 (45.3, 51.7)
Total cholesterol (mmol/l)	4.47 (4.40, 4.54)	5.15 (5.09, 5.21)	5.00 (4.94, 5.06)	5.08 (4.99, 5.18)	5.05 (4.99, 5.12)
HDL-cholesterol (mmol/l)	1.20 (1.17, 1.22)	1.27 (1.25, 1.28)	1.41 (1.39, 1.42)	1.49 (1.46, 1.53)	1.39 (1.37, 1.41)
LDL-cholesterol (mmol/l)	2.78 (2.73, 2.84)	3.38 (3.33, 3.44)	3.20 (3.16, 3.25)	3.17 (3.08, 3.24)	3.26 (3.21, 3.31)
Triacylglycerols (mmol/l)	0.97 (0.56)	0.99 (0.58)	0.73 (0.44)	0.82 (0.47)	0.76 (0.44)
Health-related behaviours					
Physical activity (MET h/week)	85 (128)	62 (152)	76 (220)	74 (190)	20 (96)
Energy intake (kJ/day)	10,847 (10,621, 11,073)	9771 (9618, 9924)	10,305 (10,058, 10,552)	12,070 (11,694, 12,447)	12,125 (11,728, 12,522)
Smoking (%)	2.3 (1.5, 3.6)	1.1 (0.6, 1.9)	4.3 (3.3, 5.6)	9.8 (7.5, 12.8)	0.5 (0.1, 1.3)
Alcohol consumption (units/week)	0.0 (0.5)	0.0 (0.2)	0.4 (1.9)	0.5 (3.5)	0.0 (0.5)

Data are presented as mean (95% CI), % (95% CI) or median (interquartile range)

BP, blood pressure

### IFBG, HOMA-IR and HOMA-B levels between geographical locations

**Fig. 2:**
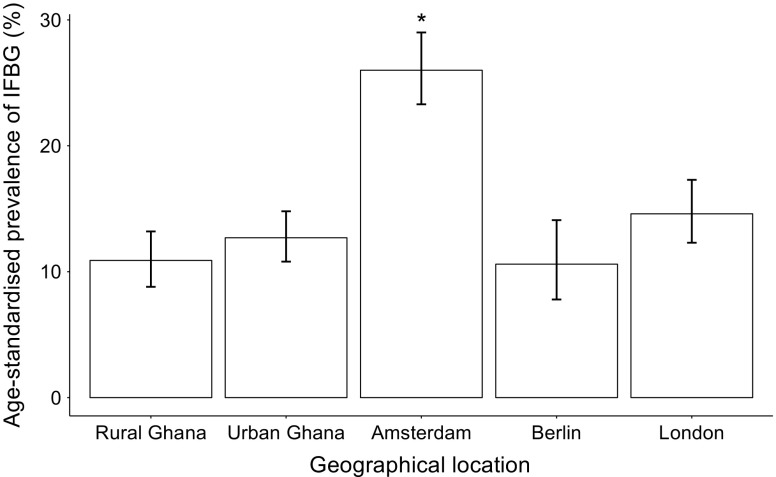
Age-standardised prevalence of IFBG per geographical location. IFBG was classified as blood glucose of 5.6–7.0 mmol/l. Data are presented as prevalence (%) ± 95% CI (*n* = 5079). *p < 0.05 vs all other locations

**Fig. 3:**
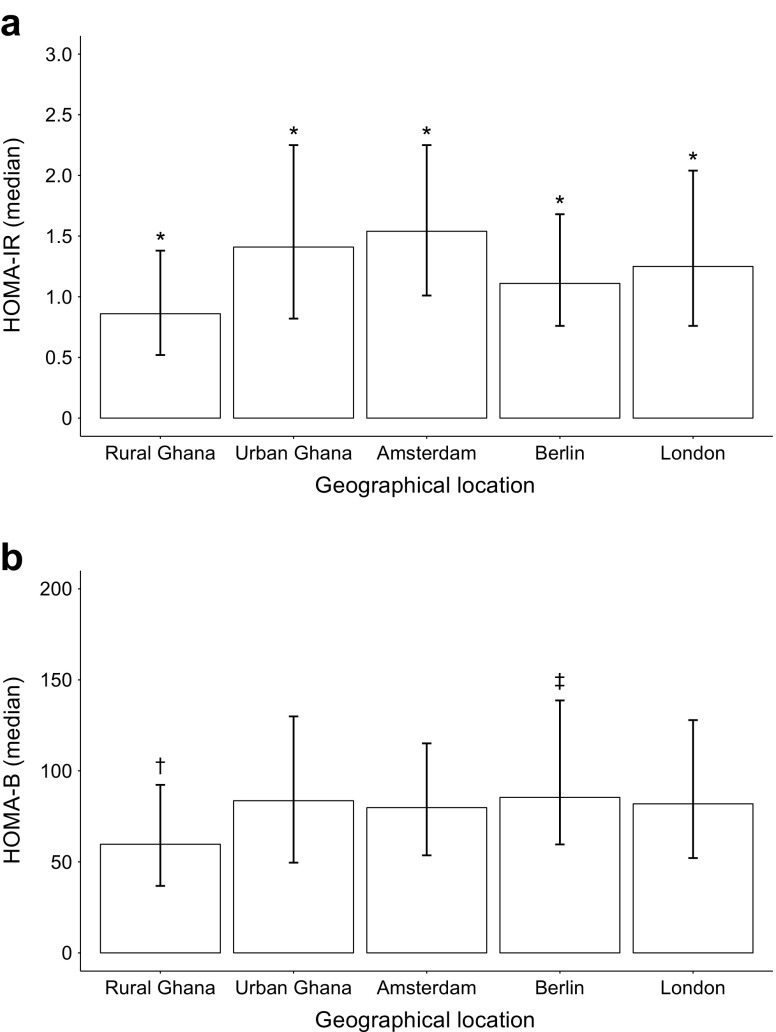
(**a**) HOMA-IR and (**b**) HOMA-B were measured in Ghanaians in different geographical locations. Data are presented as median and interquartile range (*n* = 5079). *p < 0.05 vs all other locations; ^†^ *p* < 0.05 rural Ghana vs all other locations; ^‡^ *p* < 0.05 Berlin vs urban Ghana and Amsterdam

The age-standardised prevalence of IFBG was, as expected, lower in rural Ghana (10.9%) compared with the other geographical locations, except for Berlin (10.6%) where the prevalence was similar to rural Ghana (Fig. 2). The IFBG prevalence was exceptionally high in Amsterdam (26.0%; Fig. 2). HOMA-IR differed significantly between locations (Fig. 3a); the highest median was observed in Amsterdam (1.54), while the lowest was in rural Ghana (0.86). HOMA-B was significantly lower in rural Ghana (59.7) compared with all other locations (Fig. 3b), whereas it was significantly higher in Berlin compared with urban Ghana and Amsterdam.

### HOMA-IR and HOMA-B in relation to IFBG

The age- and sex-adjusted OR for IFBG was 3.26 (95% CI 2.89, 3.69) per SD increase in HOMA-IR and 1.13 (95% CI 1.05, 1.21) per SD increase in inverse HOMA-B (Table 2). The corresponding attributable risk for IFBG was 69.3 (95% CI 63.3, 74.6) per SD increase in HOMA-IR and 11.1 (95% CI 4.9, 16.6) per SD increase in inverse HOMA-B (Table 2).

**Table 2:** Age- and sex-adjusted OR and attributable risk for IFBG per SD increase in HOMA-IR and inverse HOMA-B

	1 SD	OR (95% CI)	Attributable risk % (95% CI)
HOMA-IR	1.860	3.26 (2.89, 3.69)	69.3 (63.3, 74.6)
Inverse HOMA-B	0.015	1.13 (1.05, 1.21)	11.1 (4.9, 16.6)

In Table 3, age- and sex-adjusted ORs for IFBG are presented across the geographical locations, using rural Ghana as the reference. In addition, we assessed whether these geographical differences were accounted for by HOMA-IR or HOMA-B. Age and sex-adjusted ORs for IFBG were higher in Amsterdam (OR 3.44 [95% CI 2.69, 4.39]), London (OR 1.58 [95% CI 1.20, 2.08]) and urban Ghana (OR 1.41 [95% CI 1.08, 1.84]) compared with rural Ghana. In Berlin the odds for IFBG (OR 1.00 [95% CI 0.70, 1.45]) were similar to rural Ghana. While the ORs increased slightly when HOMA-B was included in the age- and sex-adjusted model, the inclusion of HOMA-IR reduced the ORs for urban Ghana and London so that they were similar to those for rural Ghana. For Amsterdam, the inclusion of HOMA-IR in the age- and sex-adjusted model reduced the odds for IFBG from 3.44 (95% CI 2.69, 4.39) to 2.52 (95% CI 1.94, 3.26).

**Table 3:** OR (95% CI) for IFBG per geographical location, using rural Ghana as reference

Model of adjustment	Rural Ghana	Urban Ghana	Amsterdam	Berlin	London
Age + sex	1.00	1.41 (1.08, 1.84)	3.44 (2.69, 4.39)	1.00 (0.70, 1.45)	1.58 (1.20, 2.08)
Age + sex + HOMA-IR	1.00	0.96 (0.72, 1.27)	2.52 (1.94, 3.26)	0.83 (0.56, 1.22)	1.02 (0.78, 1.38)
Age + sex + inverse HOMA-B	1.00	1.49 (1.14, 1.95)	3.76 (2.93, 4.82)	1.13 (0.78, 1.64)	1.72 (1.30, 2.27)
Age + sex + HOMA-IR + inverse HOMA-B	1.00	1.06 (0.79, 1.44)	3.30 (2.51, 4.36)	1.27 (0.84, 1.91)	1.29 (0.94, 1.77)

### Determinants of HOMA-IR and HOMA-B

In bivariate linear regression analyses, the geographical locations were strongly associated with HOMA-IR and inverse HOMA-B (Table 4). The directions of association between HOMA-IR and inverse HOMA-B were opposing for all determinants in the bivariate analyses; while female sex, family history of diabetes, higher BMI and higher waist circumference were associated with higher HOMA-IR, these determinants were associated with lower inverse HOMA-B. Besides geographical location, following the bivariate analysis the strongest positive associations with HOMA-IR were found for BMI (β = 0.45) and waist circumference (β = 0.43), and with HOMA-B, for excess alcohol consumption (β = 0.36) and smoking (β = 0.40); while the β coefficients for these determinants were reduced in the multivariate analyses, they remained strongly associated with the respective outcomes. The β coefficients for each geographical location in relation to HOMA-IR and HOMA-B were greatly reduced in the multivariate analysis because of the inclusion of all other determinants in this model.

**Table 4:** Bivariate and multivariate analyses for associations between standardised sociodemographic, anthropometric and health-related behaviour determinants with HOMA-IR and inverse HOMA-B (*n* = 3309)

Determinant	HOMA-IR		Inverse HOMA-B	
	Bivariate	Multivariate	Bivariate	Multivariate
Age	−0.07 (−0.10, −0.04)	−0.11 (−0.14, −0.08)	0.19 (0.16, 0.22)	0.20 (0.16, 0.23)
Sex (female)	0.38 (0.32, 0.43)	0.16 (0.09, 0.23)	−0.49 (−0.54, −0.43)	−0.35 (−0.42, −0.28)
Family history of diabetes	0.20 ( 0.12, 0.28)	0.12 (0.04, 0.19)	−0.14 (−0.22, −0.06)	−0.07 (−0.15, 0.02)
Anthropometrics				
BMI	0.45 (0.42, 0.47)	0.17 (0.11, 0.24)	−0.37 (−0.39, −0.34)	−0.17 (−0.24, −0.10)
Waist circumference	0.43 (0.41, 0.46)	0.29 (0.22, 0.36)	−0.32 (−0.35, −0.29)	−0.19 (−0.26, −0.12)
Health-related behaviours				
Energy intake (kJ/day)	−0.05 (−0.08, −0.02)	−0.03 (−0.06, 0.00)	0.01 (−0.02, 0.04)	0.02 (−0.02, 0.05)
Alcohol consumption^a^				
No alcohol	0.00	0.00	0.00	0.00
Within guidelines	−0.01 (−0.07, 0.05)	0.04 (−0.03, 0.10)	0.04 (−0.02, 0.10)	−0.02 (−0.08, 0.05)
Exceeding guideline	−0.30 (−0.46, −0.14)	−0.07 (−0.24, 0.10)	0.36 (0.20, 0.52)	0.25 (0.07, 0.43)
Physical activity^b^				
Low activity	0.00	0.00	0.00	0.00
Moderate activity	−0.15 (−0.24, −0.06)	−0.09 (−0.17, 0.00)	0.11 (0.02, 0.20)	0.04 (−0.05, 0.13)
High activity	−0.19 (−0.26, −0.12)	−0.12 (−0.19, −0.04)	0.17 (0.10, 0.24)	0.07 (−0.00, 0.14)
Smoking	−0.44 (−0.61, −0.28)	−0.22 (−0.41, −0.03)	0.40 (0.23, 0.57)	0.17 (−0.02, 0.36)
Geographical location				
Rural Ghana	0.00	0.00	0.00	0.00
Urban Ghana	0.55 (0.47, 0.63)	0.11 (0.03, 0.19)	−0.42 (−0.50, −0.34)	−0.01 (−0.09, 0.08)
Amsterdam	0.71 (0.63, 0.79)	0.10 (−0.00, 0.20)	−0.39 (−0.47, −0.31)	0.09 (−0.02, 0.20)
Berlin	0.35 (0.24, 0.45)	−0.07 (−0.18, 0.04)	−0.62 (−0.72, −0.51)	−0.30 (−0.41, −0.18)
London	0.50 (0.41, 0.58)	0.05 (−0.08, 0.17)	−0.50 (−0.59, −0.41)	−0.07 (−0.20, 0.05)

Data are presented as β coefficients (95% CI)

HOMA-IR and inverse HOMA-B are multiplied by 10, log transformed and converted to standardised *z* scores. All continuous determinants are also converted to standardised *z* scores

In bivariate analyses, each determinant was individually included in the model. In multivariate analyses, all determinants were simultaneously included in the model

^a^Guideline: ≤1 unit of alcohol per day for women and ≤2 units of alcohol per day for men [22]

^b^Physical activity were categorised according to MET h/week [41]

## Discussion

The findings from this study demonstrate that, compared with Ghanaians in rural Ghana, the prevalence and age- and sex-adjusted odds for IFBG were higher for those in urban Ghana, Amsterdam and London but were similar between those in rural Ghana and Berlin. Insulin resistance accounted for the differences in IFBG between locations of residence, except for Amsterdam, while beta cell dysfunction did not. BMI and waist circumference were most strongly associated with insulin resistance in Ghanaians. Exceeding the guideline for alcohol consumption and smoking were the two determinants that showed the strongest positive associations with beta cell dysfunction.

It is speculated that the increasing burden of IFBG and type 2 diabetes in African migrants and urbanising Africa might be driven by environmental exposures that disturb the balance between beta cell function and insulin sensitivity [3, 13]. To our knowledge, this is the first study that has looked at differences in insulin resistance and beta cell function among a sub-Saharan African population living in different geographical locations. Previous research by Osei et al demonstrated greater insulin resistance in African-Americans compared with Nigerians [30]. Here we found that levels of insulin resistance varied between Ghanaians living in different geographical locations, even within Europe. Insulin resistance accounted for most of the differences in IFBG between locations. In contrast, after inclusion of inverse HOMA-B in an age- and sex-adjusted logistic regression model, the odds for IFBG were increased in multiple geographical locations compared with rural Ghana. This finding is consistent with a study in Koreans that found no association between lower HOMA-B and IFBG [31]. In the current study, the lack of contribution of HOMA-B to differences in IFBG between locations and the weak association between HOMA-B and IFBG may be suggestive of compensation by the beta cells to overcome the reduced insulin sensitivity that accompanies this condition. Hence, in IFBG, environmental and associated health-related behaviour changes seem to impact insulin resistance more than beta cell dysfunction.

In sub-Saharan Africans, the prediabetes phase (defined as IFBG in this study) was suggested to be characterised by insulin resistance rather than failure of insulin secretion; per SD increase of HOMA-IR the odds for IFBG were 3.26 compared with 1.13 for 1 SD increase in HOMA-B. In the corresponding calculations of attributable risk for IFBG, we found that if the entire study population had a HOMA-IR that was 1 SD higher, there would be between 63% and 75% more individuals with IFBG. In comparison, if HOMA-B was 1 SD higher in the entire population, between 5% and 17% more individuals would have IFBG (Table 2). This is in line with studies in African-Americans that found more severe insulin resistance in individuals with normal glucose tolerance or prediabetes, the latter being defined as IFBG or impaired glucose tolerance (IGT) [8, 9, 11, 12]. On the other hand, studies among sub-Saharan Africans with type 2 diabetes have found variable insulin resistance in these individuals compared with individuals without type 2 diabetes, but fast declines in insulin secretion after diagnosis with type 2 diabetes [7, 10]. According to the model by Gibson [13], sub-Saharan Africans in a normal blood glucose state present an equilibrium characterised by both higher insulin resistance and higher beta cell function. When insulin resistance becomes more severe, initially beta cell function compensates for this change. However, when this beta cell compensation fails, type 2 diabetes develops. Longitudinal studies are needed to fully elucidate the progression from normal blood glucose state to impaired glucose tolerance to type 2 diabetes among sub-Saharan Africans.

The determinants that contributed to insulin resistance among Ghanaians included anthropometric indices that were also identified by Amoah et al in relation to IFBG in a sample of 36 Ghanaians residing in urban Ghana [32]. Additionally we found that alcohol consumption exceeding the recommendations and smoking contributed to more beta cell dysfunction. The negative associations of beta cell dysfunction with BMI and waist circumference reinforce the notion that compensation by beta cells for more severe insulin resistance might play a role in IFBG. Furthermore, a family history of diabetes was found to contribute to increased insulin resistance severity and somewhat less beta cell dysfunction. This apparent contradiction could again result from an increased compensatory beta cell function among those with more severe insulin resistance.

Lifestyle interventions, including either physical activity alone or combined with dietary advice aiming at weight loss, have been shown to significantly reduce the progression of IGT to type 2 diabetes [33]. While pharmacological intervention with, for example, metformin also targets insulin resistance, previous studies do not demonstrate it to be more effective than lifestyle interventions in terms of primary prevention [33, 34]. The reduction of BMI could be fruitful as a means to reduce the high burden of IFBG and subsequent type 2 diabetes among urban Africans and African migrants in Europe. Despite the observed associations of smoking and excessive alcohol consumption with beta cell dysfunction, public health interventions may not be worthwhile considering the relatively low prevalence of alcohol consumption and smoking in the population studied.

We found that insulin resistance accounted for the differences in IFBG between all geographical locations except Amsterdam. Among Ghanaians in Amsterdam, the odds for IFBG remained three times higher compared with rural Ghana when adjusted for insulin resistance and beta cell function. An excessively high prevalence of IFBG among Ghanaians in Amsterdam has been reported previously [35]. In addition, a very high prevalence of IFBG has also been observed among the host Dutch population [36]. We found that Ghanaians in Amsterdam had more severe insulin resistance compared with all other sites, and lower beta cell function compared with London, urban Ghana and Berlin (however, the difference in beta cell function was only significant compared with Berlin). This may imply that the failure of beta cell function to compensate for the more severe insulin resistance in Ghanaians in Amsterdam had already set in. In post hoc analyses (data not shown), waist circumference was the only modifiable risk factor associated with IFBG in Amsterdam, while in the other locations waist circumference was not associated with IFBG. Hence, there is a potential for body fat distribution and, perhaps, the ratio of subcutaneous:visceral body fat to play a role in the difference in IFBG prevalence in Ghanaians in Amsterdam compared with those in other geographical locations [37]. However, this requires further study.

It is unclear why there was a relatively low prevalence of IFBG in Berlin compared with other European sites. Previously, in the same cohort, we found Berlin to have a marginally higher prevalence of type 2 diabetes compared with other European sites and Ghana [16]. It is possible that IFBG progresses more rapidly into type 2 diabetes in this location than in other sites. Further research is required to determine the progression from normal glucose to IFBG and subsequent type 2 diabetes in different geographical contexts.

### Strengths and limitations

This was the first study to compare IFBG, HOMA-IR and HOMA-B in a large sample of relatively homogenous sub-Saharan Africans, i.e. all stemming from Ghana, resident in distinct geographical locations, using standardised approaches. We have confirmed the importance of insulin resistance as opposed to beta cell dysfunction in sub-Saharan Africans in general. Additionally, we have shown that insulin resistance is important in the context of urbanisation and migration and have studied the contribution of a range of determinants to this condition. These findings provide a foundation for future cardiovascular outcome studies, as well as studies into the progression of IFBG to type 2 diabetes in sub-Saharan Africans.

The reliance on fasting blood samples in this study only allowed us to use IFBG as an endpoint for prediabetes; thus we were not able to investigate the associations between geographical differences and determinants of IGT. While IGT and IFBG identify different groups of individuals [4], both are considered high risk groups for type 2 diabetes [38].

As a study strength, comparisons between geographical locations were unlikely to be affected by site differences as the method for fasting glucose determination was standardised between locations. Additionally, all blood samples were collected and transported according to the same protocols in each of the research locations. Biochemical analyses were performed centrally, in Charité–University Medicine Berlin, Berlin, Germany, to avoid intra-laboratory bias.

We used the geographical differences as a means to study the role of insulin resistance and beta cell dysfunction in IFBG in sub-Saharan Africans. Because of the cross-sectional nature of the data we cannot make any statements about causality. However, reverse causality between insulin resistance and IFBG, as well as between the determinants and insulin resistance, is not very likely. Nevertheless, longitudinal data are needed to study the role of environment on insulin resistance and beta cell dysfunction in the progression from normal glucose control toward IFBG and, ultimately, type 2 diabetes.

Furthermore, we used self-reported data for the identification of determinants, such as diet and physical activity. Reporting bias, such as socially desirable answering, might have influenced assessment of these determinants [39, 40]. Further in-depth investigations are needed to study the role of these health-related behaviours in IFBG among sub-Saharan Africans.

## Conclusions

We found that insulin resistance, rather than beta cell dysfunction, accounted for geographical differences in the prevalence of IFBG among Ghanaian migrants and urban Ghanaians compared with those in rural Ghana. Our results suggest that among Ghanaians, BMI and waist circumference play an important role in insulin resistance. This study was a first step to gain insight into the role of sociodemographic, anthropometric and health-related behaviour determinants in the high burden of IFBG and type 2 diabetes among African migrants in Europe and urban Africa. Longitudinal studies are needed to confirm our findings and investigate the role of other determinants in the impact of insulin resistance and beta cell dysfunction on IFBG among African populations.

## Abbreviations

HOMA-B: HOMA-derived beta cell function
IFBG: Impaired fasting blood glucose
IGT: Impaired glucose tolerance
MET: Metabolic equivalent
RODAM: Research on Obesity and Diabetes among African Migrants
